# Supplementary figures and images for: Genetic scores for predicting longevity in the Croatian oldest-old population
Source: PLoS One. 2023 Feb 3;18(2):e0279971. doi: 10.1371/journal.pone.0279971 (PMC9897585; doi:10.1371/journal.pone.0279971)

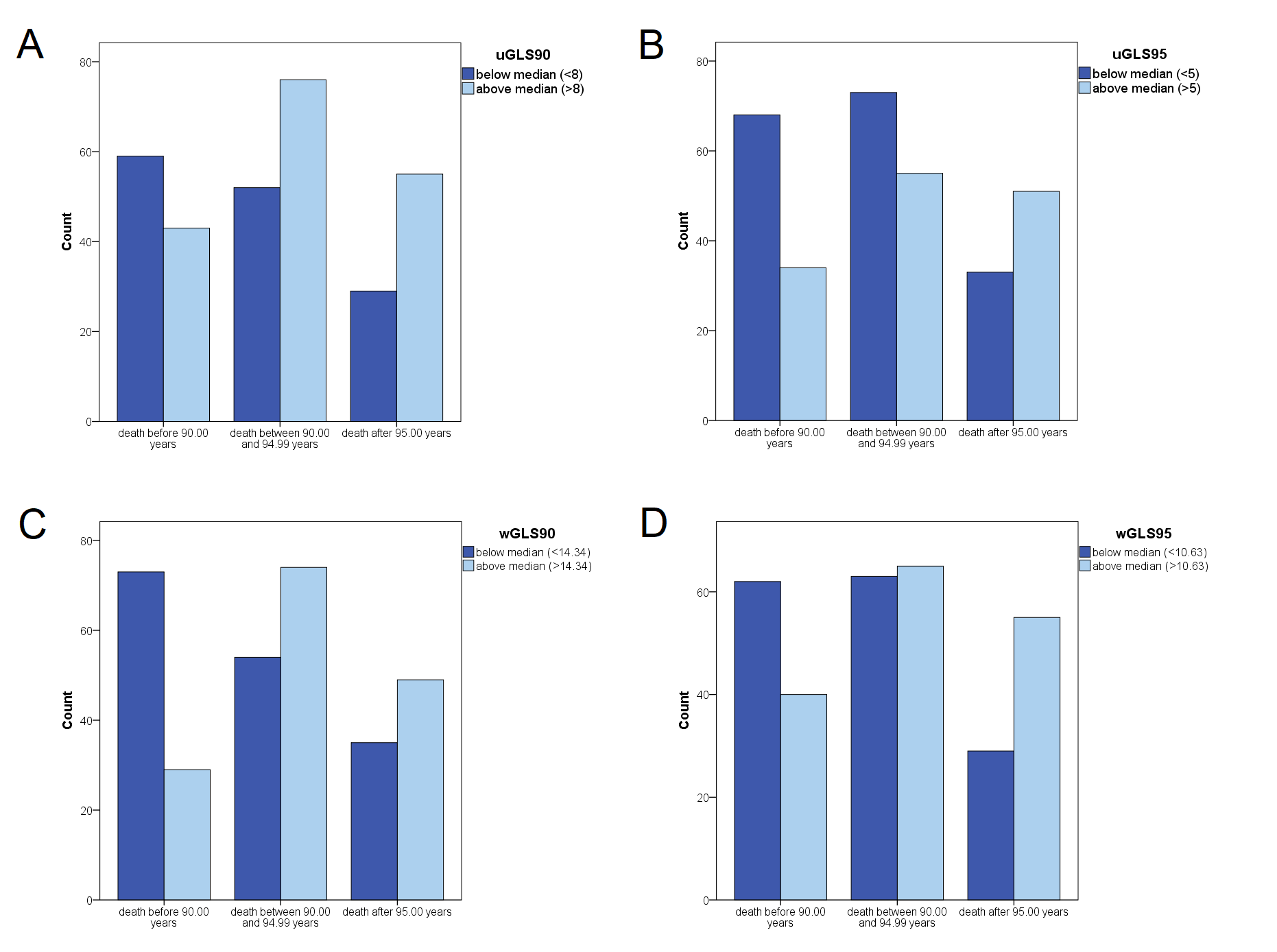

Supplement: S1 Fig — A) uGLS90, B) uGLS95, C) wGLS90, D) wGLS95. (TIF) [file pone.0279971.s004.tif]

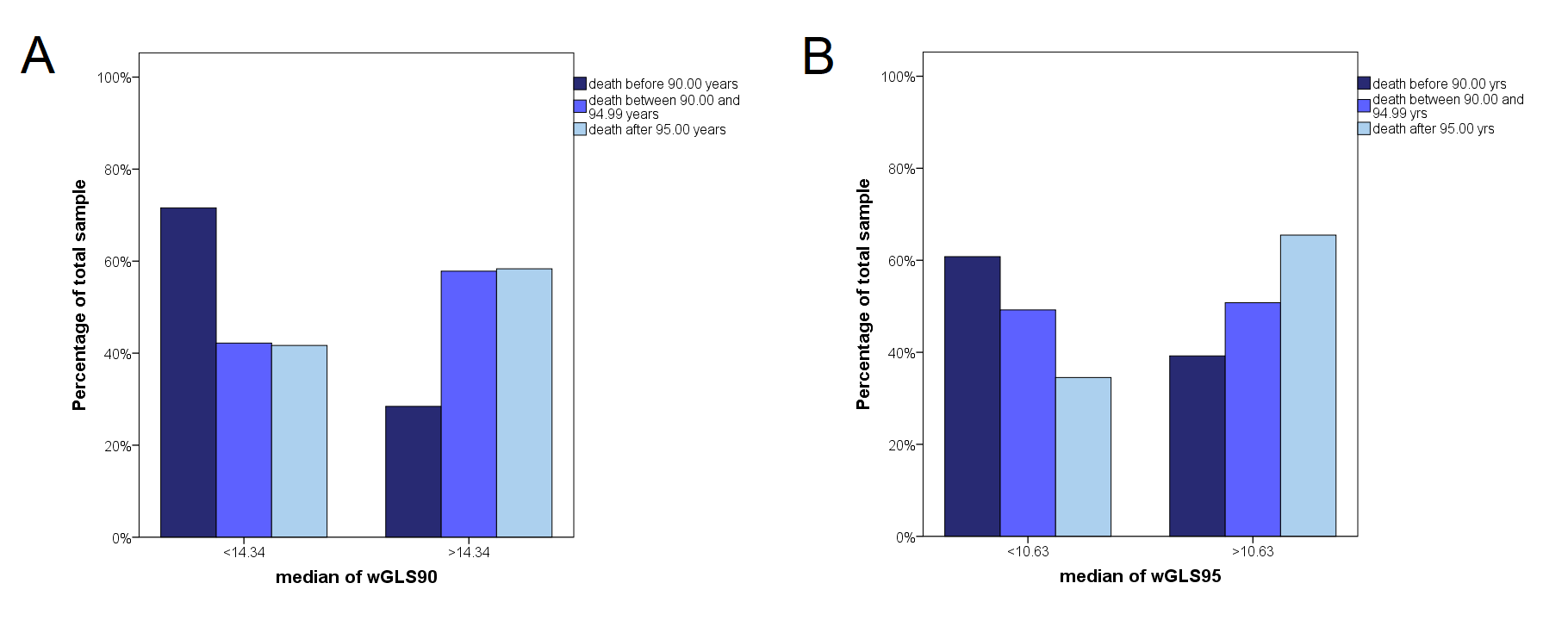

Supplement: S2 Fig — A) wGLS90, B) wGLS95. (TIF) [file pone.0279971.s005.tif]
