# Supplementary material for: Genetic scores for predicting longevity in the Croatian oldest-old population
Source: PLoS One. 2023 Feb 3;18(2):e0279971. doi: 10.1371/journal.pone.0279971 (PMC9897585; doi:10.1371/journal.pone.0279971)
Supplement: Attachment — Submitted filename: Response to Reviewers.doc [file pone.0279971.s006.doc]

Zagreb, 16 Dec 2022

Response to Reviewers

We are sincerely thankful to the Reviewer for the insightful comments and helpful suggestions. We have tried address the comments to the best of our abilities, and have made changes to the original manuscript accordingly. Below, the Reviewer’s comments are shown in blue, and our response in black.

Maja Šetinc on behalf of Authors

**Review Comments to the Author**

Reviewer #1: Šetinc and colleagues present results from a study of long-lived Croatians using targeted genotyping methods. Of the 43 variants genotyped – each with strong a priori evidence from the literature – two (2) were associated with survival to age 90+ in the multivariate model with p<0.01. In the univariate analyses, only one variant (rs2267723, GHRHR) was associated with p<0.01.

Major comments:

1. The selection of the 43 variants includes many key longevity loci from the field (APOE, SH2B3, CDKN2A, etc.) but is missing some key references. In particular the Timmers et al. 2019 meta-analysis of 500,000 people for parents’ lifespan (https://pubmed.ncbi.nlm.nih.gov/30642433) which identified several variants not included in the targeted genotyping panel. I am of course not asking you to re-do genotyping, but please acknowledge/justify this omission.

Thank You for pointing out this omission to us. We agree that this paper is very valuable to our field of research, and we have now included its mention in the text of the manuscript, along with the necessary citations. The study by Timmers et al. reports novel SNPs associated with longevity, along with validation and replication of some previous findings. Selection of SNPs associated with longevity for our study was performed in the first quarter of 2019 from a range of articles that had been published at that time, but we were, unfortunately, not aware of the paper by Timmers and colleagues at that moment. The first round of selection included over 300 possible SNPs, but due to financial constraints of our project, we had to reduce that number significantly. The main criteria for narrowing the selection were SNPs whose association has been either strong or replicated in multiple studies, and SNPs from genes involved in various pathways with well-known roles in ageing. Unfortunately, the total number of surveyed SNPs was limited to 45, and due to unsuccessful genotyping of two out of the 45, we were only able to include 43 SNPs in our analysis.

2. Table 1 (and 2) present results from a multivariate model where the genotype specification is chosen based on the best model fit parameters. This has resulted in some surprising results, for example participants heterozygous for rs1042522 located in the TP53 gene (i.e., the CG genotype group) are reported to have 1.7x likelihood of reaching 90 years, compared to the common genotype groups (CC + GG combined). Whilst there is a lot in the Discussion on p53 role in ageing/longevity the authors do not comments on why the heterozygotes only would experience lifespan benefit – a result in contract to the previous literature (which saw an additive effect with increasing G alleles).

The Arg/Arg genotype (coded by homozygous CC) induces apoptosis better than the Pro/Pro genotype (GG). This has been confirmed in multiple studies linking the Pro/Pro genotype with cancer risk. By linking apoptotic activity with a number of proliferating cells in ageing tissues, and by highlighting the need for balance between the two, we have now tried to explain why a heterozygous genotype might be more beneficial for reaching the age of 90.

2.1. I suspect that these borderline associations (p=0.04) should not be read into much, given the sample size and therefore power to detect the published effect sizes. The authors report substantially larger effects than seen in prior literature, after a running multiple iterations of the model to get the best prediction. I am not sure on the biological validity of including variants coded as HET vs HOM+HOM – normally in genetic epidemiology this format would be used to test for deviation from an additive model (i.e., justifying whether to use a recessive or dominant model).

The coding according to the recessive or dominant model (one of the homozygotes + heterozygotes) has only been used in cases where the number of participants in one of the homozygote groups was less than 10, or in two cases (rs2267723 and rs16847897) where the dominant model has proven to be more significant in the logistic regression analysis. The TP53 rs1042522 is not one of the variables that were recoded, as 14 out of the 314 participants had a GG genotype. The statistical significance of the TP53 rs1042522 in the 90-year model came from the logistic regression contrasting the relatively large number of participants with the heterozygous genotype (124 of them) against both homozygotes. And while the Exp(B) for the GG genotype suggests even higher chances of reaching 90 years of age, the effect of only 14 participants with the GG genotype was probably masked by a higher number of participants with a slightly lower, but still beneficial effect of CG. In the 95-year model, though, the GG genotype is highlighted as the significant one.

3. I personally put more belief in the univariate results available in Supplementary Table 2, where 5 SNPs were associated (p<0.05) with survival to 90+ (and one for 95+). I suggest more emphasis it put on these results in the paper as this seems better “replication” of the published effect of these SNPs. Whilst the p-values are quite borderline, each SNP has a priori evidence meaning multiple testing correction would be too conservative, and using p<0.05 is appropriate, given the analysis.

We have included a paragraph in the Results section in which we highlight the SNPs that were below the significance level of p<0.05 in the univariate analyses, and state which of these SNPs have been kept in the multivariate analysis - all except one, rs3772190, located in the *TERC* gene. The *TERC* gene was the only gene in which two of the SNPs significant on the univariate level were located. Due to linkage between the two *TERC* SNPs, rs3772190 was excluded, and rs16847897, which showed a stronger association with survival to the age of 90, was entered into the multivariate model. In order to keep the Discussion chapter as simple as possible, we chose to focus on explaining only the SNPs that were kept in the multivariate models and were used to calculate the genetic scores down the line.

4. I am actually surprised that the polygenic score is predictive of 5-year survival, given all the participants by definition reached 85 years to be eligible for the study, and the main outcome was survival to 90 years. Many of the studied “longevity” variants are actually more “disease risk” variants likely affecting mortality risk at ages below 85. There even reaching 85+ is a selected group, and this may explain why some “longevity” variants from the literature do not appear to have an effect in your study (i.e., the people who experienced the negative effects were not included?). Please discuss this point in the manuscript (what effect the participant selection had on power etc).

We agree that the pre-selection of the studied sample based on age could have some effect on the statistical power of the conducted analyses, and we have now discussed this point in the longevity score part of the Discussion section.

Minor comments:

1. Please include sample size in the abstract (N in study, % reached 90+/95+, etc).

Thank You for this suggestion; it has been included.

*

We once again want to thank the Reviewer for their expertise and for their help with improving this manuscript.
